# Cost-Effectiveness Analysis of Pembrolizumab in Patients With Advanced Esophageal Cancer Based on the KEYNOTE-181 Study

**DOI:** 10.3389/fpubh.2022.790225

**Published:** 2022-03-02

**Authors:** Mei Zhan, Ting Xu, Hanrui Zheng, Zhiyao He

**Affiliations:** ^1^Department of Pharmacy, State Key Laboratory of Biotherapy and Cancer Center, National Clinical Research Center for Geriatrics, West China Hospital, Sichuan University, Chengdu, China; ^2^West China School of Pharmacy, Sichuan University, Chengdu, China

**Keywords:** cost-effectiveness, esophageal cancer, pembrolizumab, chemotherapy, PD-L1

## Abstract

**Background and Purpose:**

The KEYNOTE-181 study demonstrated that pembrolizumab for advanced or metastatic esophageal cancer in patients with programmed death ligand-1 (PD-L1) combined positive score (CPS) ≥ 10 had a survival advantage and better tolerability than chemotherapy. However, at the same time, pembrolizumab places an economic burden on patients. This study assessed the cost-effectiveness of pembrolizumab based on the KEYNOTE181 study.

**Materials and Methods:**

A three-state Markov model [progression-free survival (PFS), progressive disease (PD), and death] based on data from the KEYNOTE-181 study was used to estimate the incremental cost-effectiveness ratio (ICER) of pembrolizumab versus chemotherapy for advanced or metastatic esophageal cancer. The model evaluates the outcomes from the Chinese society's perspective. Costs, quality-adjusted life-years (QALYs), and the ICER in terms of 2021 US$ per QALY gained, were calculated. one-way and probabilistic sensitivity analyses were performed to evaluate the model robustness.

**Results:**

Compared with chemotherapy, pembrolizumab increased costs by $37,201.68, while gaining 0.23 QALYs, resulting in an ICER of $163,165.26 per QALY in patients with PD-L1 CPS ≥ 10. The ICER is $202,708.62 per QALY and $163,643.19 per QALY in the total population and patients with esophageal squamous cell carcinoma, respectively. The ICER was much higher than the commonly accepted willingness-to-pay threshold ($11,105.8 per QALY). One-way and sensitivity analyses showed that the costs of pembrolizumab and the utility of PD were the crucial factors in determining the ICER, and probabilistic sensitivity analyses demonstrated pembrolizumab is unlikely to be cost-effective at a willingness-to-pay threshold of $11,105.8 per QALY. The result was robust across sensitivity analyses.

**Conclusion:**

Pembrolizumab is not a cost-effective treatment option for the second-line treatment of esophageal cancer from the perspective of Chinese society.

## Introduction

Esophageal cancer is a highly lethal malignancy, with 604,100 new cases and 544,076 deaths in 2020 (1). The incidence of esophageal cancer in Eastern Asia is higher when compared to the world (1, 2). China was a high-incidence area of esophageal cancer and accounted for about half of the global morbidity and mortality (3). The prognosis of esophageal cancer is improving but remains poor, with an overall 5-year survival rate of about 20% (4, 5). Approximately 39% of esophageal cancer with distant metastases at initial diagnosis and the 5-year relative survival for these esophageal cancers is about 5% (6). Although targeted drugs have demonstrated efficacy in many cancers, no targeted drugs are approved for esophageal squamous cell carcinoma. Until immune checkpoint inhibitors were approved for esophageal cancer, the treatment after first-line was limited. The goal of second-line treatment for metastatic esophageal cancer was to palliate symptoms. Unresectable, locally advanced, or metastatic esophageal cancer are not curable. Esophageal cancer contributed to a massive burden on public health care systems in China (7).

Esophageal squamous cell carcinoma is the predominant histology in China. The superiority of pembrolizumab over chemotherapy for second-line therapy in patients with metastatic esophageal squamous cell carcinoma, as well as with programmed death ligand-1 (PD-L1) combined positive score (CPS) ≥ 10 tumors, was shown in the KEYNOTE-181 study. Based on the KEYNOTE-181 study, National Medical Products Administration and the US Food and Drug Administration approved pembrolizumab for patients with locally advanced or metastatic esophageal squamous cell carcinoma that express PD-L1 (CPS ≥ 10) after one prior line in China and the USA. And the National Comprehensive Cancer Network (NCCN) recommended pembrolizumab for second-line therapy for esophageal squamous cell carcinoma with PD-L1 CPS ≥ 10 (8).

Although the KEYNOTE-181 study demonstrated a survival advantage and better tolerability for pembrolizumab, pembrolizumab is dramatically expensive for patients and insurance payers. As such, we sought to evaluate the cost-effectiveness of pembrolizumab for advanced esophageal cancer from the Chinese society perspective.

## Materials and Methods

### Patients and Regimens

The clinical information was derived from the randomized phase III, open-label KEYNOTE-181 (9). The KEYNOTE-181 study recruited adult patients with histologically confirmed, advanced, or metastatic esophageal cancer. When they progressed after first-line chemotherapy. they were randomly assigned to receive pembrolizumab 200 mg every 3 weeks or investigator's choice of treatment with paclitaxel 80–100 mg/m^2^ on days 1, 8, and 15 of each 28-day cycle, docetaxel 75 mg/m^2^ every 3 weeks, or irinotecan 180 mg/m^2^ every 2 weeks until disease progression or the development of unacceptable toxic effects. Overall survival (OS) and progression-free survival (PFS) were evaluated in patients with PD-L1 CPS ≥ 10, in patients with squamous cell carcinoma, and in the total population.

### Markov Model

**Figure 1:**
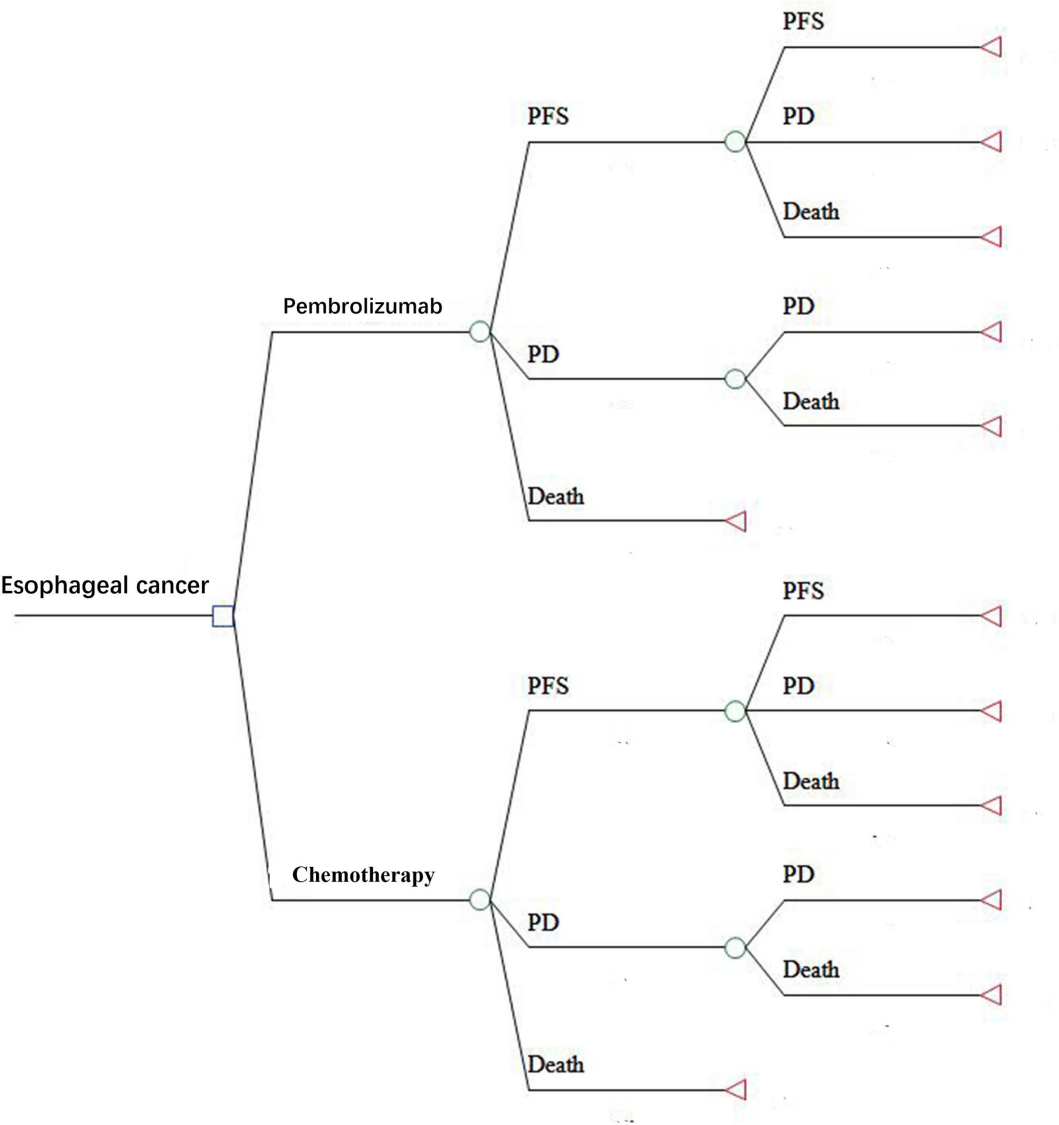
The Markov model simulated three health states: PFS, PD, and death. PFS, progression-free survival; PD, progressive disease.

A Markov model was constructed using Treeage software (Treeage, Williamstown, MA, USA) to evaluate the cost and quality-adjusted life-years (QALYs) of pembrolizumab and chemotherapy. The Markov model had three health states: PFS, progressive disease (PD), and death. The initial state was assumed to be PFS, and the patients could stay in their assigned health state or progress to another health state based on the transition probabilities during each Markov cycle. The model structure is shown in Figure 1. The cycle length was 1-month based on the time span of disease duration and progression. The 5-year survival rate was about 5% for metastatic esophageal cancer (10). Therefore, we used a 5-year time horizon in the Markov model. We calculated the incremental cost-effectiveness ratio (ICER) of pembrolizumab compared with chemotherapy, and the results are described using costs per QALY. The ICER was calculated using the following formula: ICER = (Cost_[the pembrolizumab group]_-Cost_[the chemotherapy group]_)/(QALY_[the pembrolizumab group]_-QALY_[the chemotherapy group]_). A 3% annual discount rate was used for costs and effectiveness according to the WHO guidelines (11).

### Survival Estimates and Utilities

**Figure 2:**
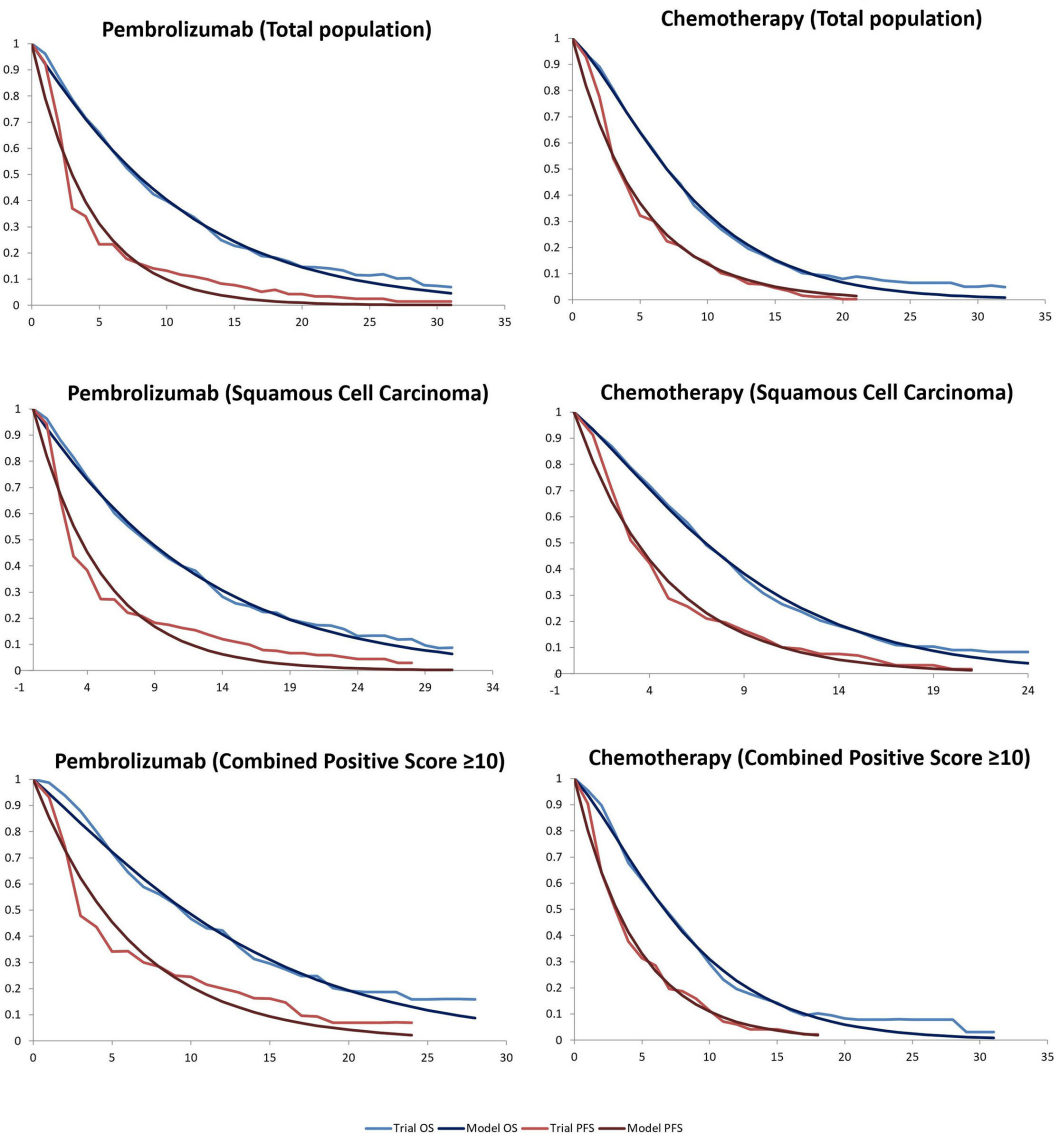
Kaplan-Meier survival for the pembrolizumab and chemotherapy groups in the KEYNOTE181 and modeled curves. OS, overall survival; PFS, progression-free survival.

Clinical data on efficacy and safety were obtained from the KEYNOTE-181 study (Table 1). The probability of being in each state was obtained by digitizing the Kaplan-Meier curves of PFS and OS from the KEYNOTE-181 study. We used Microsoft Excel (version 2016) to reconstruct the OS and PFS survival curves. Monthly transition probabilities in the reconstructed model were calibrated to best fit the Kaplan-Meier curves of OS and PFS. The monthly transition probabilities were listed in Table 2 and the calibration curves were shown in Figure 2. In the KEYNOTE-181 study, they used EORTC QLQ -C30 and QLQ-OES18, eEuroQol-5D (EQ-5D) questionnaires to assess Health-related quality of life. However, there is no information about the utility scores of the PFS and PD state in the KEYNOTE-181 study. Thus, health state utility scores were derived from previously published literature. The utility values of PFS state, PD state, and death were 0.75, 0.67, and 0, respectively (12).

**Table 1:** Basic information and base-case costs.

**Variables**	**Pembrolizumab**	**Chemotherapy**
**OS (months)**		
Total population	7.1	7.1
Combined positive score ≥ 10	9.3	6.7
Squamous cell carcinoma	8.2	7.1
**Probability of grade 3/4 AEs (%)**		
Fatigue	0.64	0.34
Decreased appetite	0.64	1.01
Asthenia	1.27	1.01
Nausea	0.00	2.36
Diarrhea	0.64	3.04
Vomiting	0.32	2.03
Anemia	1.27	7.77
Alopecia	0.00	0.34
Neutrophil count decreased	0.32	9.80
Peripheral sensory neuropathy	0.00	0.34
WBC count decreased	0.00	10.14
Neutropenia	0.00	7.09
**Cost per month($)**		
Pembrolizumab	7370.14	0.00
Chemotheapy	0.00	2231.66
Tests	916.94	727.18
Grade ≥ 3 AEs	12.23	131.79
subsequent-line therapy	150.14	150.14

**Table 2:** Transition probabilities in the Markov.

**Total population**	**Pembrolizumab**	**Chemotherapy**
pPFS-PFS	0.781	0.812
pPFS-PD	0.130	0.124
pPFS-Death	0.089	0.064
pPD-PD	0.900	0.806
pPD-Death	0.100	0.194
**Squamous cell carcinoma**		
pPFS-PFS	0.812	0.803
pPFS-PD	0.106	0.122
pPFS-Death	0.082	0.075
pPD-PD	0.911	0.832
pPD-Death	0.089	0.168
**Combined positive score ≥10**		
pPFS-PFS	0.849	0.792
pPFS-PD	0.091	0.135
pPFS-Death	0.060	0.073
pPD-PD	0.897	0.816
pPD-Death	0.103	0.184

### Cost Estimates

The direct medical costs were estimated from the perspective of Chinese society. Total costs in our analysis included costs of the drugs, tests, management of grade 3–4 adverse reactions, and follow-up. Only grade 3 or higher AEs from the KEYNOTE-181 study were used to calculate the costs of AEs, grade 1-2 AEs were considered manageable within standard patient monitoring. The cost of drugs and tests was based on the 2021 fee standards of West China Hospital, Sichuan University. We assumed that patients in the current study with a 1.72 m^2^ body surface area to calculate the dosage of chemotherapy agents (13–15). After the disease progressed, the data of patients who received subsequent treatment was insufficient from the KEYNOTE-181 study. Hence, the costs of subsequent-line therapy were derived from a previously published study (16). The details of the cost information are provided in Table 3. The WHO guidelines recommended 1–3 times the Gross Domestic Product per capita (GDP) as the threshold of willingness to pay (WTP) (17). As a result, $11,105.8/QALY was set according to the per capita GDP of China 2020 released by the National Bureau of Statistics. All costs were converted into US dollars, with an exchange rate of $1 = ¥6.4831 (August 11, 2021).

**Table 3:** Cost parameters input in the model.

**Parameter**	**Value($)**
**Drug acquisition**	
Pembrolizumab 100 mg	2763.80
Paclitaxel 30 mg	104.89
Docetaxel 20 mg	200.52
Irinotecan 100 mg	273.50
**Laboratory tests and scans**	
12-Lead Electrocardiogram	16.20
PT/INR and Aptt	6.79
Chemistry panel	23.29
Routine blood tests	2.31
Urinalysis	4.94
T3, FT4, and TSH	47.35
Echocardiography	37.79
Chest and abdomen CT	389.63
Other	377.44
Bed fee/day	6.32
**SAE management cost per event**	
Fatigue or Asthenia	214.43
Decreased appetite	105.57
Nausea or Vomiting	98.83
Anemia	328.90
Peripheral sensory neuropathy	17.49
WBC or Neutropenia count decreased	356.31

### Sensitivity Analysis

One-way sensitivity analysis was performed by varying key parameters within ± 20% of its baseline value individually to examine the potential influence on the results. Probabilistic sensitivity analysis (PSA) was conducted with 10,000 Monte Carlo simulations. Gamma distribution was selected for the cost parameters, beta distribution was selected for utility. The results of the PSA were represented by cost-acceptance curves. If the ICER was below the threshold of WTP, interventions were defined as cost-effective.

## Results

### Base-Case Analysis

In the base-case analysis, for the whole population, the total costs of the pembrolizumab group were $34,272.16, and the total costs of the chemotherapy group were $15,217.55. The overall QALYs in the Pembrolizumab group were higher than that in the chemotherapy group (0.57 vs. 0.48 QALYs). The ICER of the pembrolizumab group compared with the chemotherapy group was $202,708.62 per QALY, which was almost 6 times higher than the WTP threshold for cost-effectiveness ($11,105.8 per QALY in China). Subgroup analysis showed that pembrolizumab gained the most QALY in the subgroup with PD-L1 CPS ≥ 10. Pembrolizumab cost $37,201.68 more than chemotherapy and provided additional.23 QALYs, leading to an ICER of $163,165.26 per QALY in patients with PD-L1 CPS ≥ 10. When pembrolizumab was administered in patients with squamous cell carcinoma, the ICERs were $163,643.19 /QALY, in comparison with chemotherapy. The ICERs were beyond the WTP thresholds in all subgroups, demonstrating that pembrolizumab was not a cost-effective strategy for patients with advanced or metastatic esophageal cancer from the Chinese perspective. The details are listed in Table 4.

**Table 4:** Results of the cost-effectiveness analysis.

**Subgroups and strategies**	**Total population**		**Combined positive score ≥10**		**Squamous cell carcinoma**	
	**Pembrolizumab**	**Chemotherapy**	**Pembrolizumab**	**Chemotherapy**	**Pembrolizumab**	**Chemotherapy**
**Costs ($)**						
PFS state($)	33412.67	14718.98	50050.34	13173.71	39525.06	13985.27
PD state($)	859.46	498.57	842.62	517.55	909.93	539.81
Total cost ($)	34272.16	15217.55	50892.96	13691.28	40434.99	14252.08
Incremental cost ($)	19054.61	/	37201.68	/	26182.91	/
**Effectiveness(QALYs)**						
PFS state (QALYs)	0.25	0.30	0.38	0.27	0.30	0.28
PD state (QALYs)	0.32	0.18	0.31	0.19	0.34	0.20
Total effectiveness (QALYs)	0.57	0.48	0.69	0.46	0.64	0.48
Incremental effectiveness (QALYs)	0.09	/	0.23	/	0.16	/
ICERs compared with PC alone ($/QALY)	202708.62		163165.26		163643.19	

### Sensitivity Analyses

**Figure 3:**
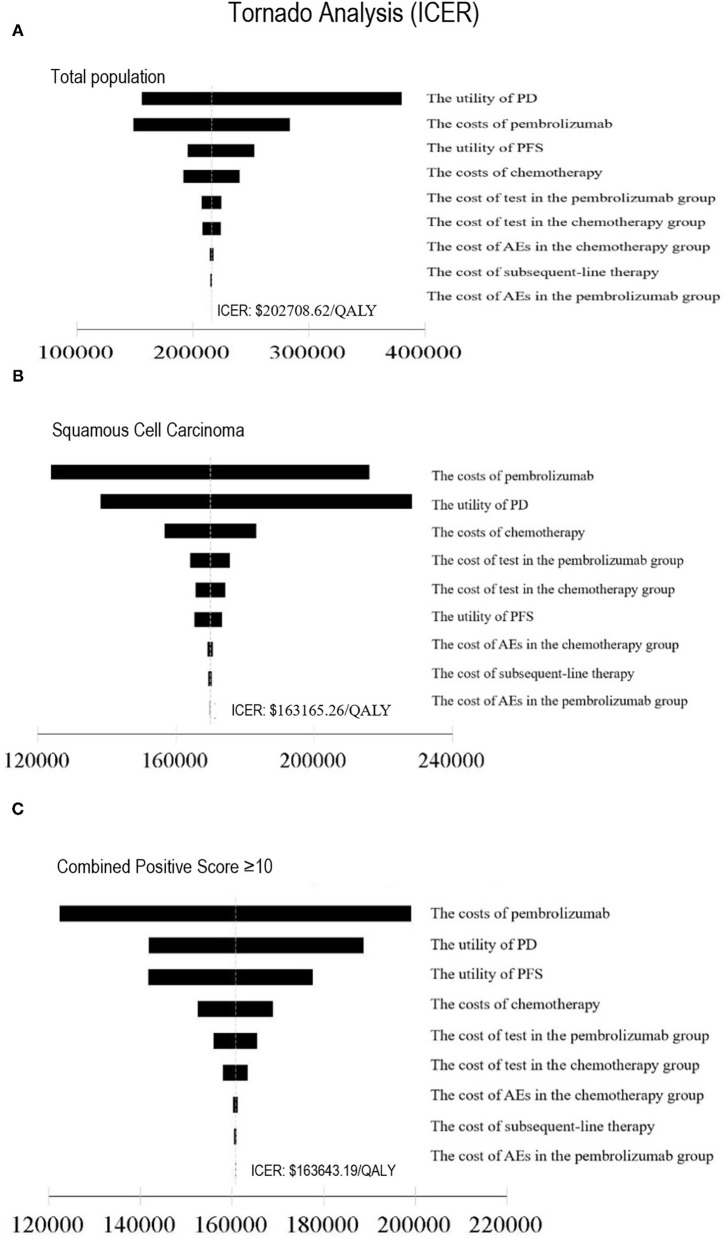
Tornado diagram of one-way sensitivity analysis. This summarizes the results of one-way sensitivity analysis, listing influential parameters in descending order according to their effect on the ICER over the variation of each parameter value. ICER, Incremental Cost-Effectiveness Ratio; **(A)** Total population, **(B)** Squamous Cell Carcinoma, **(C)** Combined Positive Score ≥10, PFS, progression-free survival; PD, progressive disease; AE, adverse event.

**Figure 4:**
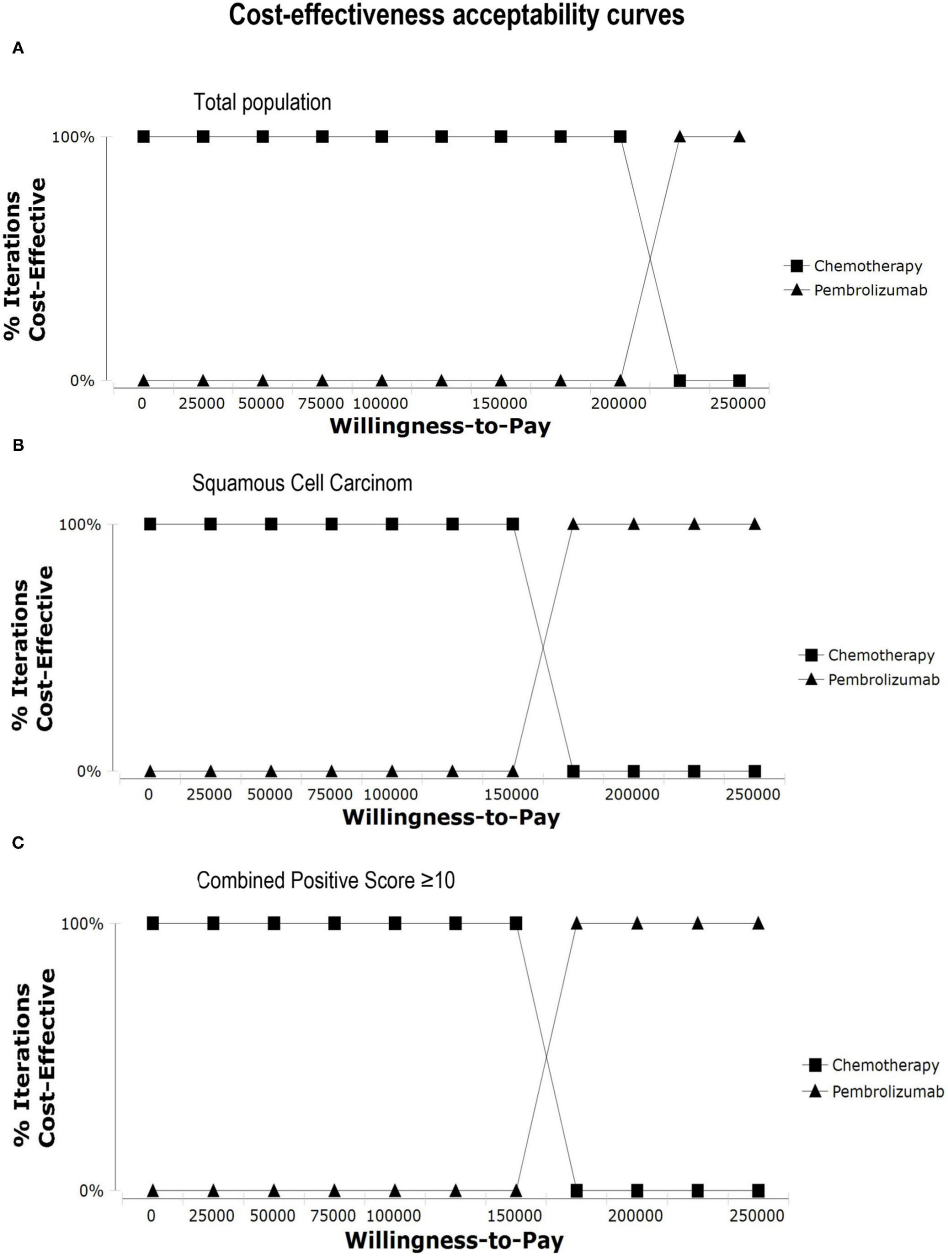
Cost-effectiveness acceptability curves. Cost-effectiveness acceptability curves show the probability of each treatment strategy being cost-effective at different WTP thresholds. **(A)** Total population, **(B)** Squamous Cell Carcinoma, **(C)** Combined Positive Score ≥ 10.

In one-way sensitivity analysis, for different subgroups, the most influential factor on the results was different. The price of pembrolizumab had the highest impact on the ICER for patients with PD-L1 CPS ≥ 10 or squamous cell carcinoma of the esophagus. For the total population, the utility of PD had the most influential factor in our model, but the price of pembrolizumab also had a great impact on the robustness of the cost-effectiveness analysis. The one-way sensitivity analyses revealed that the price of pembrolizumab and the utility of PD was the most sensitive model input. Other variables, such as the utility of PFS, the costs of chemotherapy and test had a moderate impact on the results, and the cost of AEs and subsequent-line therapy had a minimal impact on the outcome. Changing individual parameters did not change the results, pembrolizumab had no chance to be cost-effective at the current WTP threshold. More details of one-way sensitivity analyses were depicted in Figure 3. The results of probability sensitivity analyses for different subgroups are shown in Figure 4. With the increasing WTP value, the acceptable proportion of the chemotherapy group was decreased, whereas the pembrolizumab group was increased. However, pembrolizumab had no possibility of being a cost-effective treatment unless the threshold of cost-effectiveness analysis sharply increased to about $175,000 per QALY. And it seems that China's GDP cannot reach this level in the short term.

## Discussion

In this study, we found that pembrolizumab was not cost-effective for advanced or metastatic esophageal cancer compared with chemotherapy. The price of pembrolizumab had the highest impact on the ICER when pembrolizumab was administered in patients with squamous cell carcinoma or PD-L1 CPS ≥ 10. Because of the assistance programs for esophageal cancer, patients with squamous cell carcinoma and PD-L1 CPS ≥ 10 can buy two cycles of pembrolizumab and obtain two cycles of pembrolizumab free. When we calculated the cost of pembrolizumab according to the donation plan, the ICER dropped to $65,122.91 per QALY and $53,955.88 per QALY in patients with PD-L1 CPS ≥ 10 and squamous cell carcinoma, respectively. Although pembrolizumab provided the greatest clinical benefit in QALYs for patients with PD-L1 CPS ≥ 10, the cost of PFS state was less in the esophageal squamous cell carcinoma due to the shorter PFS compared with the PD-L1 CPS ≥ 10. The KEYNOTE181 study showed that the greatest survival benefit was observed in patients with squamous cell carcinoma and PD-L1 CPS ≥ 10. But the Supplementary Appendix of the KEYNOTE-181 study only provided the OS curve, not the PFS curve in patients with squamous cell carcinoma PD-L1 CPS ≥ 10 tumors, we cannot estimate the probability of being in each state based on the PFS and OS curves. We did not conduct the Markov model to assess the cost-effectiveness of pembrolizumab in patients with squamous cell carcinoma PD-L1 CPS ≥ 10 tumors.

PD-1 and PD-L1 inhibitors have become an effective treatment strategy for advanced esophageal cancer (18–21), and have been increasingly used after first-line therapies in advanced esophageal cancer (22). Pembrolizumab prolonged survival in patients with many solid cancers (23), but due to the high price of pembrolizumab, the economics of pembrolizumab is controversial (24). Factors including clinical effectiveness, safety, and drug price had an impact on the results of the pharmacoeconomic evaluation. Treatment options are limited for metastatic esophageal cancer, especially after first-line treatment. The survival benefit of Chemotherapy is modest and the side effects of chemotherapy reduce the quality of life. Immunotherapy using immune checkpoint inhibitors, such as nivolumab, pembrolizumab, and camrelizumab, has resulted in survival benefits and fewer side effects for patients with metastatic esophageal cancer. The study performed a Markov model to assess the cost-effectiveness of nivolumab vs. chemotherapy in the second-line treatment for advanced esophageal squamous cell carcinoma based on the ATTRACTION-3 trial. The results of the study showed that nivolumab was not a cost-effective treatment option compared with chemotherapy from the Chinese society perspective (25). Because of the sharp price reduction of camrelizumab, the pharmacoeconomic research results of camrelizumab as second-line therapy for advanced or metastatic esophageal squamous cell carcinoma were inconsistent (14, 15, 26), Yang et al. (14) showed camrelizumab was not cost-effective as second-line therapy for advanced or metastatic esophageal squamous cell carcinoma compared with chemotherapy in China. The cost of camrelizumab in Yang et al. was $2,802 per 200 mg. Both Lin et al. (26) and Cai et al. (15) showed camrelizumab was cost-effective compared with chemotherapy for advanced or metastatic esophageal squamous cell carcinoma (15, 26). The price of camrelizumab was only $452.87 per 200 mg and $432 per 200 mg in Lin et al. (26) and Cai et al. (15), respectively. Up to date, there has been no cost-effectiveness analysis of pembrolizumab as second-line therapy for advanced esophageal cancer.

WTP is a critical parameter to determine whether the intervention is cost-effective. If the ICER was lower than the WTP, the intervention is considered to be favorably cost-effective. The WTP was set as 1–3 times GDP per capita according to the WHO guidelines that were widely referenced in the last decade (27). But some studies suggest that three times of GDP per capita as WTP is too high and the WTP threshold is below 1 × GDP per capita (28, 29). The National Institute of Health and Clinical Excellence (NICE) proposed that the cost-effectiveness threshold of life-extending treatments for patients with terminally ill patients could increase to £50,000/QALY (30, 31) The WTP threshold used in Greek cost-effectiveness studies showed that the median WTP threshold used in oncology studies [€51,000 (€50,000–57,000)] was higher than in non-oncology studies [€34,000 (€30,000–€35,000); *p*-value < 0.001] (32). There is no established standard in China for the WTP. According to the guidelines and literature, 1 × GDP per capita was set as WTP in our study. The research has certain limitations associated with model-based cost-effectiveness analyses. We primarily relied on the data from a phase III trial rather than real-world experience. Occasionally, real-world experience may deviate from that seen in trials. In addition, detailed data on the clinical and economic burden of treatment-related adverse events remain limited. First, the KEYNOTE181 study only reported the OS and PFS in patients with PD-L1 CPS ≥ 10 tumors, squamous cell carcinoma, and in all patients, respectively, but reported the adverse events in all patients. We hypothesized that the incidences of AEs were similar between different subgroups and estimated the cost of AEs according to the safety outcomes in the total population. Second, the cost data in the model were obtained from official or published prices, but the cost of drugs and tests varied across different regions in China. Third, nutrition support plays an important part in the management of esophageal cancer. As this part of the information was not mentioned in the KEYNOTE-048 trial, these costs were not considered. Besides, due to the lack of utility data in China, the utility values were obtained from Western countries. Imprecise estimates and assumptions were necessary and this uncertainty was evaluated using sensitivity analysis. The results of sensitivity analyses with a range of ± 20% of variation showed that the results were stable.

China has launched national reforms on centralizing drug procurement to contain drug costs. A series of Chinese domestic PD-1 inhibitors including camrelizumab, sintilimab, toripalimab, tislelizumab were included in medical insurance with a price reduction of more than 64%. With the development of the drug industry, more and more PD-1 inhibitors will emerge, which might provide an alternative for patients with esophageal squamous cell carcinoma in China and promote the price reduction of pembrolizumab. With the price adjustment of PD-1 inhibitors, the economy of these drugs will be improved.

## Data Availability Statement

The original contributions presented in the study are included in the article/Supplementary Material, further inquiries can be directed to the corresponding author.

## Ethics Statement

Ethical review and approval was not required for the study on human participants in accordance with the local legislation and institutional requirements. Written informed consent for participation was not required for this study in accordance with the national legislation and the institutional requirements.

## Author Contributions

MZ participated in research design, data processing, and manuscript writing, TX and HZ in data processing. ZH participated in research design and manuscript revising. All authors contributed to the article and approved the submitted version.

## Funding

This work was supported by the 1.3.5 project for disciplines of excellence-clinical research incubation project, West China Hospital, Sichuan University (2021HXFH064).

## Conflict of Interest

The authors declare that the research was conducted in the absence of any commercial or financial relationships that could be construed as a potential conflict of interest.

## Publisher's Note

All claims expressed in this article are solely those of the authors and do not necessarily represent those of their affiliated organizations, or those of the publisher, the editors and the reviewers. Any product that may be evaluated in this article, or claim that may be made by its manufacturer, is not guaranteed or endorsed by the publisher.
